# 5-Aminolevulinic acid enhances cancer radiotherapy in a mouse tumor model

**DOI:** 10.1186/2193-1801-2-602

**Published:** 2013-11-12

**Authors:** Junko Takahashi, Masaki Misawa, Mami Murakami, Takashi Mori, Kazuki Nomura, Hitoshi Iwahashi

**Affiliations:** Biomedical Research Institute, National Institute of Advanced Industrial Science and Technology, Tsukuba, Ibaraki, Japan; Human Technology Research Institute, National Institute of Advanced Industrial Science and Technology, Tsukuba, Ibaraki, Japan; Faculty of Applied Biological Sciences, Gifu University, Gifu, Japan

**Keywords:** 5-aminolevulinic acid, Cancer, Protoporphyrin, Radiotherapy, X-ray

## Abstract

5-Aminolevulinic acid (ALA) is a photosensitizer used in photodynamic therapy (PDT) because it causes preferential accumulation of protoporphyrin IX (PpIX) in tumor cells, where it forms singlet oxygen upon light irradiation and kills the tumor cells. Our previous study demonstrated that PpIX enhances generation of reactive oxygen species by physicochemical interaction with X-rays. We investigated the effect of ALA administration with X-ray irradiation of mouse B16-BL6 melanoma cells *in vitro* and *in vivo*. ALA facilitates PpIX accumulation in tumor cells and enhances ROS generation *in vitro*. Tumor suppression significantly improved in animals treated with fractionated doses of radiation (3 Gy × 10; total, 30 Gy) with local administration of 50 mg/kg ALA at 24 h prior to fractional irradiation. These results suggest ALA may improve the efficacy of cancer radiotherapy by acting as a radiomediator.

## Background

In cancer therapy, radiotherapy is preferred to surgical resection because it is non-invasive, allowing organ structures and functions to remain intact. Radiotherapy damages the DNA of cancerous cells by direct or indirect ionization of the atoms that make up the DNA chain. However, tumor responses to radiation vary with repair capacity, oxygenation, and other factors, and side effects in normal tissues increase with the higher doses used to kill radio-resistant tumor cells Peters et al. (1982). To increase the sensitivity of the tumor site alone, many potential radiosensitizers have been studied (Rotman et al. 1998). The mechanism of most radiosensitizers, except oxygen or nitric oxide, involves inhibition of nucleic acid synthesis, angiogenesis, DNA repair, and cell signaling, eventually inducing apoptosis in treated cells. To control and limit irradiation to tumor cells, sparing normal cells, it is important to develop methods to enhance dosing precision.

Photodynamic therapy (PDT) is used to treat certain cancerous and pre-cancerous dermatological conditions. It is preferred over surgical resection because it is non-invasive, as is the case for radiotherapy. PDT, established in the 1970s, is based on the interaction of light with photosensitive agents known as photosensitizers that preferentially accumulate in target cells and produce energy transfer and a local chemical effect (Ding et al. 2011). After exposure to specific wavelengths of light, the photosensitizer is excited from the ground state to the singlet state, then undergoes type I (electron transfer) and/or type II (energy transfer) reactions to produce reactive oxygen species (ROS), resulting in necrosis and/or apoptosis of exposed cells Pass (1993). The penetration depth of the light source though tissue limits treatment to tumors on or under the skin, or on the lining of some internal organs, because successful use of PDT requires light activation of photosensitizers.

Protoporphyrin IX (PpIX) were examined as a candidate PDT photosensitizer with biological compatibility and measured the species and amount of ROS generated by X-ray irradiation (Takahashi & Misawa 2009). ROS detection reagents (APF and DHE) and ethanol quencher were used in solutions containing different concentrations of PpIX in the study. This combination of experimental conditions allowed us to estimate the contribution of PpIX to the generation of hydroxyl radical (∙OH), superoxide anion (O_2_^-^), and singlet oxygen (^1^O_2_). Today, 5-aminolevulinic acid (ALA)-PDT is used to treat a variety of neoplastic and non-neoplastic disorders to avoid the prolonged photosensitivity caused by systemic administration (Peng et al. 1997). ALA, a heme precursor, acts as a pro-drug and is absorbed and converted by the heme biosynthetic pathway to photoactive PpIX, which accumulates preferentially in rapidly dividing cells after light exposure, without cytotoxicity. We hypothesized that PpIX accumulation could be induced by adding exogenous ALA, and through its generation of ROS by X-ray irradiation in cancer cells, may act as a radio-mediator of radiotherapy. This study investigated the effect of ALA administration with X-ray irradiation of mouse B16-BL6 melanoma cells *in vitro* and *in vivo*.

## Methods and materials

### Chemicals

ALA hydrochloride, methanol, perchloric acid, acetic acid, sodium hydrogen carbonate, RPMI1680 medium, penicillin, streptomycin, fetal bovine serum, and PBS were purchased from Wako Chemicals (Osaka, Japan). CellROX™ Deep Red Reagent was from Invitrogen (Carlsbad, CA).

### Cell culture

The B16-BL6 mouse melanoma cell line was supplied by the Riken Cell Bank (Tsukuba, Japan) and cultured in PRMI1640 (GIBCO BRL, Life Technologies, Tokyo, Japan) containing 10% FBS (Moregate BioTech, QLD, Australia) in a 5% CO_2_ humidified incubator at 37°C. The medium was supplemented with 100 units/mL penicillin and 100 μg/mL streptomycin (GIBCO).

### Determination of porphyrin concentration in cells and tissue

Porphyrin in cells or tissue samples was isolated in 1.0 N perchloric acid and methanol (1:1 v/v) after homogenization and centrifugation at 3000 rpm for 10 min. The supernatant was transferred to a tube, neutralized with sodium hydrogen carbonate, and acidified with acetic acid (96%). Porphyrin concentration was determined by spectrophotometry at the Soret maximum (405 nm) and fluorescence using an excitation wavelength of 405 nm and an emission wavelength of 630 nm (Doss & Schmidt 1971). Presentation of tissue data per gram-wet weight was performed as described previously.

### Measurement of intracellular ROS

We performed *in vitro* studies to estimate the effect of ALA and X-ray irradiation on intracellular ROS in B16-BL6 cells. The cell-permeable CellROX™ Deep Red dye is nonfluorescent in the reduced state; upon oxidation, it exhibits excitation/emission maxima at 640/665 nm. B16-BL6 cells were cultivated in 96-well plates to confluence. ALA was added 24 h before X-ray irradiation in 100 μL of culture medium at 1, 5, 10, 50, and 100 μg/mL. Control cells were incubated without ALA. Before X-ray irradiation, CellROX™ Deep Red Reagent was added at a final concentration of 10 μM. The plates were placed 0.46 m from the x-ray generator and irradiated for 1.0, 3.0, 5.0, and 10.0 min, corresponding to 1.0, 3.0, 5.0, and 10.0 Gy absorbed dose. After irradiation, plates were incubated for 30 min at 37°C. The medium was removed and the cells were washed with PBS. Fluorescence was measured on a microplate reader (Infinite M200, TECAN).

### Animal studies

The general procedure for the mouse B16-BL6 mouse melanoma model was described previously (Jin et al. 2005). Briefly, 6-week-old female C57BL/6 J mice purchased from Charles River Laboratories Japan, Inc. (Yokohama, Japan) were used for all experiments. Mice were subcutaneously injected with B16-BL6 cells (1.3 × 10^5^ cells) in 0.1 mL medium without FBS or antibiotics.

Mice were randomized into 4 groups (n = 5, each group) after implantation of B16-BL6 cells; (1) control group; (2) ALA treatment; (3) X-ray treatment; (4) ALA and X-ray treatment. After 3 d, mice in the X-ray and ALA and X-ray treatment groups were irradiated with 3 Gy daily q.d. (quaque die) × 5 × 2 weeks, for a total dose of 30 Gy. Mice in the ALA and X-ray treatment group were administrated local ALA diluted in PBS at 50 mg/kg bodyweight 24 h before X-ray irradiation. The mice in the ALA treatment group received ALA at the same time. Tumor volume, based on caliper measurements, was calculated every 10 days according to the following formula: tumor volume = the shortest diameter^2^ × the largest diameter × 0.5 (Jin et al. 2005).

### X-ray irradiation conditions

A polychromatic, diagnostic X-ray generator (KXO-15E, Toshiba Medical Systems Corp., Tochigi, Japan) was operated at a tube voltage of 100 kV and a tube current of 4 mA. In vivo study, a mouse was held tight in a plastic holder with an opening above the tumor area. The collimated X-ray beam irradiated a 24 × 24 mm area at the tumor site, large enough to cover the entire area of the maximum tumor. A free air ionization chamber (RAMTEC1500-DC300, ToyoMedic Ltd., Tokyo, Japan) was used for dose rate measurement. The resulting dose rate was 1.007 Gy/min at the sample stage.

### Statistics

Accumulation of porphyrin in B16-BL6 cells, intracellular ROS were analyzed by two tailed Student’s t-test. Tumor volume changes and body weight were analyzed by one-way analysis of variance. The Tukey-Kramer HSD test was used for post-hoc pair-wise comparison. Differences were significant at P < 0.05.

### Ethical considerations

All experimental protocols were approved by the Committee for the Care and Use of Experimental Animals at AIST (Permit Number: 2012–097).

## Results and discussion

### ALA uptake kinetics of B16-BL6 melanoma cells in vitro and in vivo

**Figure 1:**
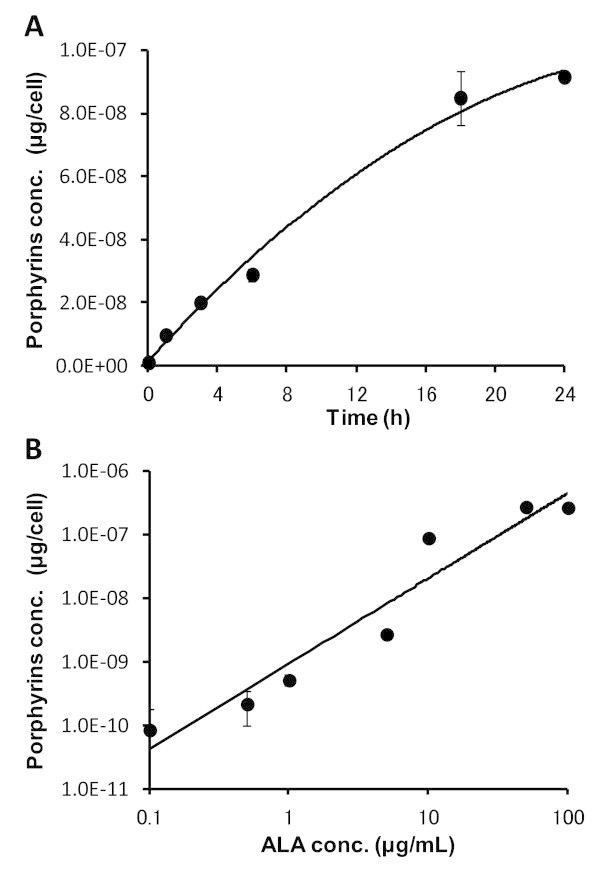
**Kinetics of total porphyrin levels in B16-BL6 mouse melanoma cells** ***in vitro*** **. A**, Cells were incubated with 10 μg/mL ALA. **B**, Cells were incubated with different concentrations of ALA for 24 h. Data are given as mean ± SD (n = 4).

ALA that has entered the cytoplasm may enter the heme synthesis pathway and transiently accumulate PpIX. PpIX converted from ALA preferentially accumulates in tumors, the accumulation depending on the kind of tumor or administration method. To estimate the behavior of ALA in B16-BL6 cells *in vivo*, porphyrin levels were examined *in vitro*. Although other kinds of porphyrin were detected by this method, PpIX is the major porphyrin in cells or tissues (Fritsch et al. 1997). Figure 1A shows porphyrin kinetics in B16-BL6 cells incubated with 10 μg/mL ALA. Porphyrin levels increased with incubation time. Figure 1B shows the porphyrin concentration in B16-BL6 cells incubated with ALA for 24 h. The porphyrin concentration was plotted as a function of ALA concentration. Porphyrin accumulation increased with increasing ALA concentration in B16-BL6 cells after 24 h incubation *in vitro*.

ALA has been approved as a photosensitizer for PDT in the USA and Europe, and its pharmacokinetics, toxicity, and accumulation in cancer are well understood (Zhao & He 2010). In photodynamic therapy (PDT) the photo-sensitizer 5- ALA can be used by systemic, oral or topical application. 5-ALA is administrated systemically at some clinical trials on use of PDT for advance cancer (Brown et al. 2004). After treatment with ALA, PpIX preferentially accumulates in tumor cells; excitation by light brings PpIX to the singlet state, which emits fluorescence upon returning to the ground state. Therefore, ALA has been used for fluorescence-guided surgery or photodynamic diagnosis by systemic or local administration. Lofgren et al. reported the optimal treatment time is 3–6 h post-ALA intravenous injection of 100–200 mg/kg for rabbits in PDT, because the porphyrin ratio between papilloma and normal skin was highest, providing optimized efficiency without risk of significant damage to normal skin (Lofgren et al. 1995).

We used a protocol in which ALA was administered immediately after X-ray irradiation for convenient preparation for the next irradiation; tumor porphyrin was measured 24 h after ALA subcutaneous intratumoral injection (50 mg/kg). Porphyrin accumulation in implanted B16-BL6 tumors 24 h after administration was 6.2 times higher with local than with systematic, local administration providing efficiencies equal or surpassing that of PDT (3.0 ± 1.4 μg per gram wet weight). This report is the first trial to confirm the effect of ALA administration with X-ray and to study the story in vivo. The practical procedures for therapy have to be studied in the future.

### ROS induction by ALA and X-ray irradiation in vitro

**Figure 2:**
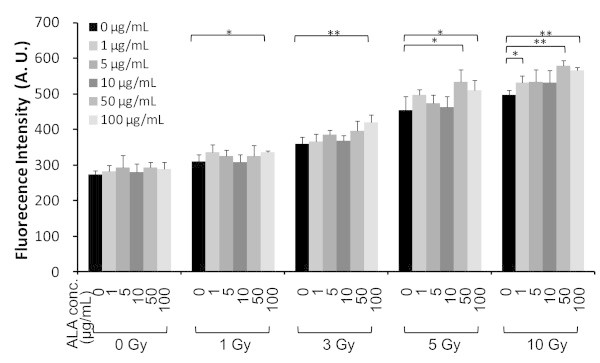
**Intracellular ROS level viability of ALA and X-ray treatment.** Intracellular ROS level of B16-BL6 mouse melanoma cells with various concentrations of ALA and different X-ray doses *in vitro*. ALA was added 24 h before X-ray irradiation. Before X-ray irradiation, CellROX™ Deep Red Reagent was added at a final concentration of 10 μM to the cells. After X-ray irradiation, plates were incubated for 30 min at 37 degree. Subsequently, medium was removed and the cells were washed with PBS. The resulting fluorescence was measured using a microplate reader. Data are given as mean ± SD (n = 4). An asterisk indicates significant difference compared to without ALA. *P < 0.05, **P < 0.01 for ALA treatment vs. the untreated control.

*In vitro* studies were performed to estimate the effect of ALA and X-ray treatment on intracellular ROS generation in B16-BL6 cells with CellRox™ Deep Red dye. CellROX™ Deep Red Reagent is a fluorogenic probe designed to reliably measure ROS in living cells. ROS level is expressed in reference to the non-irradiated control plate. Figure 2 shows ALA effects on intercellular ROS level at different X-ray doses. B16-BL6 cells were incubated with ALA for 24 h before X-ray irradiation. As a control, cells without ALA were irradiated under the same conditions.

Intracellular ROS increased with X-ray dose and ALA concentration (Figure 2). Student’s t-test results showed a significant difference between the control and 1 μg/mL ALA treatments at 10 Gy X-ray irradiation, between the control and 50 μg/mL ALA treatments at 5 and 10 Gy X-ray irradiation, and between the control and 100 μg/mL ALA treatments at 1, 3, 5 and 10 Gy X-ray irradiation. In this study, PpIX enhanced ROS generation induced by X-ray irradiation in addition to those by radiolysis.

### In vivo tumor suppression by ALA and irradiation

**Figure 3:**
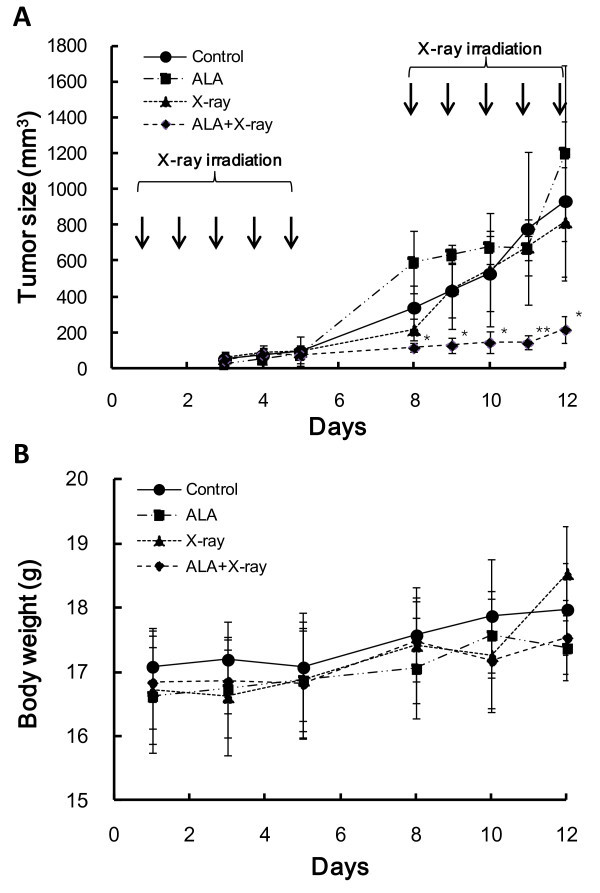
**ALA potentiates B16-BL6 tumor suppression by X-ray irradiation.** C57Bl/6 mice bearing B16-BL6 cells were as follows: (1) control; (2) ALA; (3); irradiation, 10 locally fractionated doses of 3 Gy each; (4) irradiation after ALA treatment, 10 locally fractionated doses of 3 Gy each. Data are given as means ± SD (n = 5, *P < 0.05 vs. control, **P < 0.01 vs. control). Tumor size **(A)** was measured daily and body weight **(B)** was measured 3 times a week; curves were plotted up to day 12.

To evaluate tumor suppression by ALA treatment with radiotherapy *in vivo*, a C57BL/6 J melanoma tumor model was used. ALA was administrated as a local dose of 50 mg/kg 24 h before X-ray irradiation. Figure 3A illustrates tumor growth during fractionated doses of irradiation. B16-BL6 tumors were resistant to radiation, as the ALA-only and irradiation-only groups did not show a tumor suppression effect. However, the fractionated doses of X-ray irradiation after ALA intratumoral injection significantly inhibited tumor growth 8 d after treatment initiation. ALA synergistically sensitized B16-BL6 tumors to radiation without systemic toxicity (Figure 3B).

Although the mechanism by which PpIX induces ROS generation after X-ray irradiation remains unclear, we propose the following model. Under X-ray irradiation, the ionizing photons yield primary radicals as ∙OH, ∙H, and e-aq during water radiolysis. Scattered photons with lower energies also generate secondary electrons and cause excitation of triplet oxygen. These energies are transferred to PpIX and raise it to an excited state. During de-excitation, PpIX may generate O_2_^-^ and ∙OH, as suggested in previous study (Takahashi & Misawa 2009). Therefore, the primary sites of action of ALA-PDT may be restricted to the sites of PpIX production and/or accumulation; many studies have described the localization and yield of PpIX in many tumor types (Berg 2001; Herman et al. 1997). However, these studies addressed only tumors of the skin or within the penetration depth of a given light source. ALA-X-ray radiotherapy enables application in tumors deep under the skin or in body organs or invasive cancers that are difficult to remove surgically due to the large x-ray penetration depth.

## Conclusions

In conclusion, ALA improved tumor suppression by X-ray irradiation *in vivo*, as PpIX accumulated in tumor cells. The X-ray dosing conditions and ALA concentrations have been well characterized in PDT treatment.

## Abbreviations

ALA: 5-aminolevulinic acid
PDT: Photodynamic therapy
PpIX: Protoporphyrin IX
ROS: Reactive oxygen species.
